# Referential Choices in a Collaborative Storytelling Task: Discourse Stages and Referential Complexity Matter

**DOI:** 10.3389/fpsyg.2018.00176

**Published:** 2018-02-20

**Authors:** Marion Fossard, Amélie M. Achim, Lucie Rousier-Vercruyssen, Sylvia Gonzalez, Alexandre Bureau, Maud Champagne-Lavau

**Affiliations:** ^1^Faculté des lettres et sciences humaines, Institut des Sciences du Langage et de la Communication, Université de Neuchâtel, Neuchâtel, Switzerland; ^2^Centre de Recherche CERVO, Québec City, QC, Canada; ^3^Département de Psychiatrie et Neurosciences, Université Laval, Québec City, QC, Canada; ^4^Département de Médecine Sociale et Préventive, Université Laval, Québec City, QC, Canada; ^5^Centre National de la Recherche Scientifique UMR 7309, LPL Aix-Marseille University, Aix-en-Provence, France

**Keywords:** storytelling, referential choices, referential complexity, visual salience, discourse, accessibility, collaboration, interaction

## Abstract

During a narrative discourse, accessibility of the referents is rarely fixed once and for all. Rather, each referent varies in accessibility as the discourse unfolds, depending on the presence and prominence of the other referents. This leads the speaker to use various referential expressions to refer to the main protagonists of the story at different moments in the narrative. This study relies on a new, collaborative storytelling in sequence task designed to assess how speakers adjust their referential choices when they refer to different characters at specific discourse stages corresponding to the introduction, maintaining, or shift of the character in focus, in increasingly complex referential contexts. Referential complexity of the stories was manipulated through variations in the number of characters (1 vs. 2) and, for stories in which there were two characters, in their ambiguity in gender (different vs. same gender). Data were coded for the type of reference markers as well as the type of reference content (i.e., the extent of the information provided in the referential expression). Results showed that, beyond the expected effects of discourse stages on reference markers (more indefinite markers at the introduction stage, more pronouns at the maintaining stage, and more definite markers at the shift stage), the number of characters and their ambiguity in gender also modulated speakers' referential choices at specific discourse stages, For the maintaining stage, an effect of the number of characters was observed for the use of pronouns and of definite markers, with more pronouns when there was a single character, sometimes replaced by definite expressions when two characters were present in the story. For the shift stage, an effect of gender ambiguity was specifically noted for the reference content with more specific information provided in the referential expression when there was referential ambiguity. Reference content is an aspect of referential marking that is rarely addressed in a narrative context, yet it revealed a quite flexible referential behavior by the speakers.

## Introduction

Reference is a fundamental act in language communication. In our everyday conversations, we exchange and share information about different things—people, objects, and events—present in the linguistic or situational context, and we do so using a complex referential system based on a large variety of linguistic expressions. Answering the question “how do people refer to ‘things”’ is far from being obvious. Firstly, there are multiple theoretical conceptions, with a continuum of views on the referential act ranging from “addressee-blind or at least addressee-myopic […] to a cooperative, or coordinated, act that requires speakers to consider their addressees” (p. 45, Clark and Bangerter, 2004). Secondly, there is no simple or “one to one” correspondence between one referent and one expression. The same female individual, for instance, can be mentioned in very different ways, going from highly informative forms, such as the full indefinite noun phrase (NP) “*a tall blond girl with a hat,”* to less explicit forms, such as the pronoun “*she.”* Furthermore, during a continuous discourse or conversation, the cognitive status of the referents is rarely fixed once and for all. Rather, each referent varies in accessibility as the discourse progresses, being influenced for instance by the presence and prominence of other entities, leading the speaker to constantly readjust his referential choices depending on the level of cognitive accessibility of the targeted referent. In this paper, we will examine how speakers adjust their referential choices when they refer to more or less accessible entities in referential contexts that increase in complexity, due to the number and gender ambiguity of the referents in the visual display. To this end, our analyses will not only focus on the choice of reference markers (indefinite marker, definite marker, or pronoun) but also on reference content (i.e., the extent of the information provided in the NP), two complementary characteristics of referential expressions that have however rarely been jointly studied.

Most referential theories assume that the choice of any particular referential expression is closely connected to the accessibility or salience that the referent is assumed to have, at a given moment, in the discourse representation (e.g., Givon, 1983; Ariel, 1990, 2001; Gundel et al., 1993; Chafe, 1994; Gundel, 1998). Factors that can affect the accessibility of an entity are numerous and heterogeneous (resulting from different sources, linguistic and non-linguistic), making the notion of accessibility complex and irreducible to any single factor (Ariel, 2001; Arnold, 2010). At the linguistic level, different discourse features have been identified to affect salience. In particular, a referent that is *given* (i.e., previously evoked in the discourse) and *topical* in the discourse (i.e., recently mentioned, especially in a syntactically prominent position like the subject) is generally more accessible (Ariel, 1990; Gordon et al., 1993; Chafe, 1994; see also Arnold, 2010; Arnold et al., 2013). While the topical referent is considered to be the most salient in the discourse—in the “focus of attention”—(Grosz et al., 1995), “given” referents may not always be highly accessible, leading to a gradient of “givenness.” There exists a well-established consensus that the more salient or accessible a referent is, the more reduced and attenuated the expression used by the speaker, and conversely, the less salient or accessible a referent is, the more elaborate or explicit the referential expression (Arnold, 2010). Following Ariel's accessibility marking scale (Ariel, 1990) or Gundel et al.'s givenness hierarchy (Gundel et al., 1993), unaccented pronouns (and zeros) are particularly expected to refer to highly accessible referents, whereas a large range of “intermediate” expressions, including accented pronouns, demonstratives, and definite NPs, are more expected for referents with a “medium” (and possibly low) accessibility level. While Ariel did not discuss the use of indefinite references, these markers are typically used to introduce new referents that were thus not already accessible in the discourse context. This idea is in line with Gundel et al.'s claim (Gundel et al., 1993, 2012) that an indefinite determiner only requires that the referent be type-identifiable (i.e., identifiable as a member of its category).

In a narrative context, once the referent has been introduced it can then be mentioned again at different points in the discourse, and different studies indicate that referential forms can vary from one mention to the next. To elicit narratives for stories involving several protagonists (referents), different visual supports (without text) can be employed including cartoon videos (e.g., Arnold et al., 2009) or picture booklets (e.g., Hickmann et al., 1995; Van der Lely, 1997; Colle et al., 2008; Hendriks et al., 2014; Kuijper et al., 2015; Contemori and Dussias, 2016). This allows to examine the referential expressions used to refer to the main protagonists of the stories at different moments in the storytelling. Several languages (English, but also Dutch and French) were investigated in these studies, typically focusing on the choices of reference markers.

For instance, Arnold et al. (2009) analyzed the frequency at which pronouns were produced according to how recently the same character (i.e., referent) had previously been mentioned and in which syntactic position (subject or non-subject) in narratives relating to a cartoon video. The results indicated that speakers used pronouns more often when referents had been mentioned recently (i.e., mentioned in the immediately preceding clause) or prominently (i.e., in subject position). In particular, as the number of clauses increased following the last mention of the referent, the use of pronouns decreased, achieving <10% when the referent had not been mentioned at all. Essentially, as the salience of the referent decreased, the use of pronouns decreased as well, a result likely linked to the fact that the discourse status of the referent then changes, no longer being in the focus of attention (i.e., was no longer the most prominent discourse referent at that point).

Others, such as Colle et al. (2008) used a 24 picture booklet and distinguished three moments or discourse stages in the produced narratives, namely the introduction of characters being referred to for the first time (a boy and a dog in their story), the reintroduction of the characters after a different character had been referred to, and the maintaining of the reference to a character in subject or object position. Results for the healthy adult group revealed a clear referential pattern with a general preference for introducing the characters with indefinite NPs (compared to definite NPs), more “nominals” (full NPs category mixing indefinite and definite NPs) to reintroduce the characters, and conversely, more “pronominals” (including pronouns and zero anaphors) to maintain the references.

A recent storytelling task by Hendriks et al. (2014) confirmed this overall pattern but also highlighted that other factors can modulate this effect of the discourse stages. In the task used by Hendriks et al. (2014), participants were asked to tell picture-based stories featuring two characters of the same gender to a hypothetical listener. The six pictures of each story were shown one at a time. The first and second pictures displayed the first character only. A second character entered the story in the third picture, and in the fourth and fifth pictures he performed an action. The final picture showed again the first character alone. Hendriks et al. (2014) analyzed the references to the two characters at five positions in the story: Introduction and maintaining of first character, introduction and maintaining of second character, and re-introduction of first character^1^. In young adults, their results showed an important use of full NPs (“nominals”) at the introduction stage (for each of the two characters) as well as at the re-introduction stage, examined for character 1 only and defined as his re-introduction after the second character had been mentioned). At the maintaining stage, the choice of referential form depended on the character being referred to. More specifically, pronouns were massively used when maintaining references to the first character (i.e., defined as the next reference to the first character immediately after his introduction) but not to the second character, for which full NPs were more often used. This seemingly over-informative referential behavior (i.e., using full NPs for what the authors designated as the maintaining of character 2) could yet indicate that the second character, at this point in the discourse was not the most salient referent because, for instance, it was mentioned in a syntactically less prominent position (a non-subject position) in the previous utterance. As proposed by Hendriks et al. “in some narratives the speaker may not yet have clearly established the second character as the new topic” (p. 404, Hendriks et al., 2014), which could explain the use of NPs instead of pronouns to bring these characters in focus. In other words, the “Maintain-2” position in Hendriks et al.'s study suggests a rather floating discourse status of the referent (i.e., the second character)^2^, sometimes already established as the new topic (i.e., in the focus of attention) but only for a part of the cases. There are thus important variations in speakers' referential choices, which could be explained by the storybook images where the second character is never displayed as the most visually salient character. Yet, visual salience is an important feature that can influence the choice of the referent that is mentioned first as the subject of the utterance (Vogels et al., 2013).

The influence of the visual context on speakers' referential choices in discourse situations has been relatively little investigated. The effect of visual salience is well established when there is no prior linguistic context (e.g., Osgood, 1971; Parkhurst et al., 2002; Mazza et al., 2005), but the role of visual information in the referential process during discourse remains to be better understood. Recently, story completion experiments highlighted different effects of the visual context on referential choices when a linguistic context is present, depending on which kind of information is manipulated in the visual scene (e.g., number of possible referents, visual salience of the referents). For instance, Arnold and Griffin (2007) asked participants to continue stories illustrated in a two-panel picture. Each story was primed with a context sentence describing the first panel and participants were asked to generate the next sentence based on the second panel. The stories staged one or two characters, of different genders. Arnold and Griffin found that participants produced fewer pronouns to refer to the main character (i.e., the character “in the focus of attention”) when another character (the competitor) was also present. This was true even when the competitor was present only in the first panel and absent in the second panel, indicating that it is not the visual presence of the competitor in the second panel—during sentence generation—that was the source of this effect. Instead, it seems to be the presence of the competitor in the preceding discourse (the context sentence) and/or the preceding image (the first panel) that reduced the use of pronouns.

In order to further explore this competition effect and understand the role of visual information on speaker's referential choice, Fukumura et al. (2010) used a similar experiment in which both the linguistic and visual contexts were manipulated in terms of the presence or absence of a competitor character. While Arnold and Griffin had only manipulated the visual presence of the competitor in the second picture (second panel), Fukumura et al. manipulated the presence of the competitor character in both pictures. They found that the visual presence of the competitor reduced pronoun use, and that this effect was even larger when the competitor had been linguistically mentioned than when it was not. Interestingly, the fact that even when the competitor was not linguistically introduced, its visual presence reduced pronoun use, suggests that “the competitor can become part of the discourse representation even though it has not been linguistically mentioned” (p. 1706, Fukumura et al., 2010). These findings thus show that speakers take visual information into account when choosing a referential expression (but see also Kantola and Van Gompel, 2016, for evidence that this may be specific to conditions in which a real addressee is present).

Beyond the visual presence of a competitor, and thus the number of possible referents in the scene, other features of the visual context, such as the relative visual salience of the referents, can play a role in referent accessibility. Recently, Vogels et al. (2013) investigated the effect of the relative visual salience of two characters, and its interaction with the linguistic context, both on the choice of the subject referent and on the choice of referential expression. In their study, the two characters were of different gender and their relative visual salience was manipulated by having one of the characters in the foreground of the picture while the other was in the background. The agentivity of the characters was also manipulated with one of the characters performing a simple action (agent character) while the other was passive (non-agent character). The results indicated that participants referred to the agent character as the subject of their utterances in the vast majority of the cases, achieving up to 98% of the references when the agent character was visually salient. References for the non-agent character being too few in number to conduct statistical analyses, only the results for the agent character were reported by the authors. The results indicated that the visual salience influenced the choice of referent but not the choice of referential expression. The visually foregrounded character was indeed more likely to be chosen and referred to first, as the subject of the sentence, but it was not more likely to be pronominalized, suggesting that different processes are at work in choosing a subject referent and choosing a referential expression. As expected, the use of pronouns depended on the referent's linguistic salience, that is, whether the referent was the subject of the previous sentence (cf. Gordon et al., 1993; Grosz et al., 1995; Arnold et al., 2009; Arnold, 2010), but this choice was not influenced by the referent's visual salience.

In sum, manipulating the visual context in terms of the relative visual salience of the referents (foregrounding) in the scene, as in Vogels et al's study (2013), led to different results to those of Fukumura et al. (2010), for instance, who only manipulated the number of possible referents (1 vs. 2). The number of referents and the relative visual salience of the referents thus appear as two dimensions of visual context that can differently affect speaker's referential choices. In the current study, these two dimensions were taken into account to develop original cartoon sequences used to elicit storytelling. In each of the six images forming the cartoon sequences, one of the characters is both more agentive and more visually salient than the other. Following Vogels et al. (2013), this manipulation should directly influence the choice of the character to refer to first, as the subject of the sentence. We thus expect that focusing our analyses on the visually salient, agent character in each of the images will lead to a clear distinction between three discourse stages, namely the introduction of the referent, its maintaining in focus (corresponding to the second consecutive time the character is active and in the foreground in the picture), or to the shift of the referent who is in focus (corresponding to the character moved to the foreground in the picture and who becomes active). This new way of methodologically defining the three discourse stages (introduction, maintaining, and shift of the referent in focus) should provide a better match to the conceptual definitions of these discourse stages and facilitate, therefore, the analysis of the references, preventing unclear discourse status of the referents. In addition, manipulating the number (1 vs. 2) and gender (same vs. different) of the possible referents within the same task will allow us to establish different levels of referential complexity for the stories, which will likely influence the choices of referential expressions.

Remarkably, the studies cited above uniquely focused on the choices of reference markers, and they limited their analyses to the single contrast between pronouns vs. full NPs. However, there is numerous evidence that speakers also adjust the content of their referential expressions by adding one or more modifiers to the head noun of their NPs (typically definite NPs), particularly when the target referent is accompanied by another referent of the same type (Davies and Katsos, 2016). Several studies reported that when speakers are asked to present a target object (e.g., a candle) to an addressee who has to identify it within a set of objects, speakers use modified definite expressions (e.g., “the red candle” or “the small candle”) when multiple possible referents are present that are visually ambiguous, allowing the object to be uniquely identified (Ferreira et al., 2005; Brown-Schmidt and Tanenhaus, 2006; Engelhardt et al., 2006; Koolen et al., 2011). Sometimes, speakers also generate modified expressions even though they know that their addressee is not aware of the contrasting object (Horton and Keysar, 1996; Nadig and Sedivy, 2002; Lane and Ferreira, 2008), a finding that has been interpreted as indicating the influence of an egocentric bias in the process of language production. However, including more information in a description than strictly necessary does not jeopardize communication (in this case, referent identification for the addressee). It would rather be to provide too little information—being referentially under-specific—that may be seen as reflecting an egocentric tendency, detrimental for communication (see also Achim et al., 2015).

Interestingly, providing “too much information” seems to be quite frequent in verbal communication (Davies and Katsos, 2016). For instance, Pechmann (1989) reported results from a language production experiment in which speakers were asked to describe an object among a set of distractor objects, and they found that over 20% of referential expressions were overinformative, that is, containing at least one redundant attribute (e.g., color or size). In a recent study, Koolen et al. (2011) found that around 50% of referential expressions were overspecified, and Engelhardt et al. (2006) showed that even in single-referent conditions (in which the target referent is displayed without other referents of the same type), modified descriptions occurred in 30% of referential expressions, a surprisingly high rate for only one possible referent, but lower than in the two-referent condition (98%). Essentially, it appears that speakers are fairly likely to over-describe the referents, while they consistently avoid under-descriptions, with only 5% of the references that were underspecified in Koolen et al.'s study. While quite interesting, all these studies that investigated reference content, in particular over-specification, did so during target identification tasks only. Whilst it is likely that people also adjust the content of their referential expressions during a continuous discourse, it remains to be objectively established. But in order to do so, it is crucial to take into account the effect of the discourse stages (introduction, maintaining, or shift of the referent in focus), given that the referent's accessibility varies at different points in the discourse, which needs to be taken into account.

### Research objectives and hypotheses

The present study aimed to simultaneously examine the effect of discourse stages and of referential complexity of the stories on speakers' referential choices using a collaborative discourse task. Two main objectives were followed. The first objective was to examine whether French-speakers adapt their choices of reference markers (indefinite marker, definite marker, or pronoun) based primarily on discourse stages (introduction of a new referent; maintaining, and shift of the referent in focus), and if so, if this adjustment is observed for stories with different levels of referential complexity, namely low referential complexity (stories with 1 character), intermediate referential complexity (stories with 2 characters of different gender), and high referential complexity (stories with 2 characters of the same gender). We hypothesized that an important adjustment would be observed in the use of referential expressions as narrative elaboration goes along, with more indefinite markers for the introduction stage, more pronouns for the maintaining stage, and more definite markers for the shift stage. This pattern was expected to be strong and observed for all levels of referential complexity.

Our second objective was to investigate whether, within a given discourse stage, finer variations either in the use of reference markers or in the content of the referential expressions could be observed as a function of the levels of referential complexity of the stories. Referential complexity can increase either when there is an increased number of characters or when two (or more) characters have the same gender, and both of these factors were manipulated in this study leading to three levels of referential complexity (see section Methods for more details). Our main expectation was that an increased referential complexity of the stories should lead to more explicit or more elaborate referential expressions, permitting to identify the intended referent more precisely. However, the use of more elaborate referential expressions could take different forms depending on the discourse stages. For instance, at the maintaining stage where pronouns are highly expected (see hypothesis above), a more explicit expression could take the form of an increased use of definite expressions (e.g., “the girl” instead of “she,” when the referential complexity increases). But at the shift stage, where definite expressions are already expected to be the most often used, a more explicit expression could take the form of a more specific reference content (e.g., “the blond girl” vs. “the girl”).

In sum, while analyzing the various types of reference markers allows us to examine the dynamics of referential adjustment through different discourse stages, analyzing reference content is interesting in particular, to investigate the effect of referential complexity of the stories on the amount of information provided in the NP. Currently, little is known about how people adjust their choices of referential expressions in discourse when they refer to more or less accessible entities in increasingly complex referential contexts. The current study thus targets this research gap while examining both reference markers and reference content.

## Methods

### Participants

Thirty participants were recruited from the community in the region of Neuchâtel, Switzerland. They were all native French speakers and aged 18–37 years old (mean age = 24.9; 24 men; mean education = 14 years). Participants were excluded if presenting cerebral or neurologic antecedents, psychiatric disorders, or if having important uncorrected vision or audition impairments. The local ethics boards of the University of Neuchâtel approved the study and all participants signed an informed consent in accordance with the Declaration of Helsinki to participate in the study.

### Material and procedure

#### The story telling in sequence task

A new storytelling in sequence task was developed and optimized specifically for this study. The task comprises nine narrative sequences, each composed of six colored images (size: 10 × 11.5 cm) displaying 1 or 2 characters in everyday life situations. The material, initially designed by Courchesne et al. (2009), allows us to manipulate and study the effect of two parameters of interest, namely the referential complexity of the story (between sequences: 3 levels) and the discourse stages (within sequences: 2 or 3 stages).

##### Levels of referential complexity

**Figure 1:**
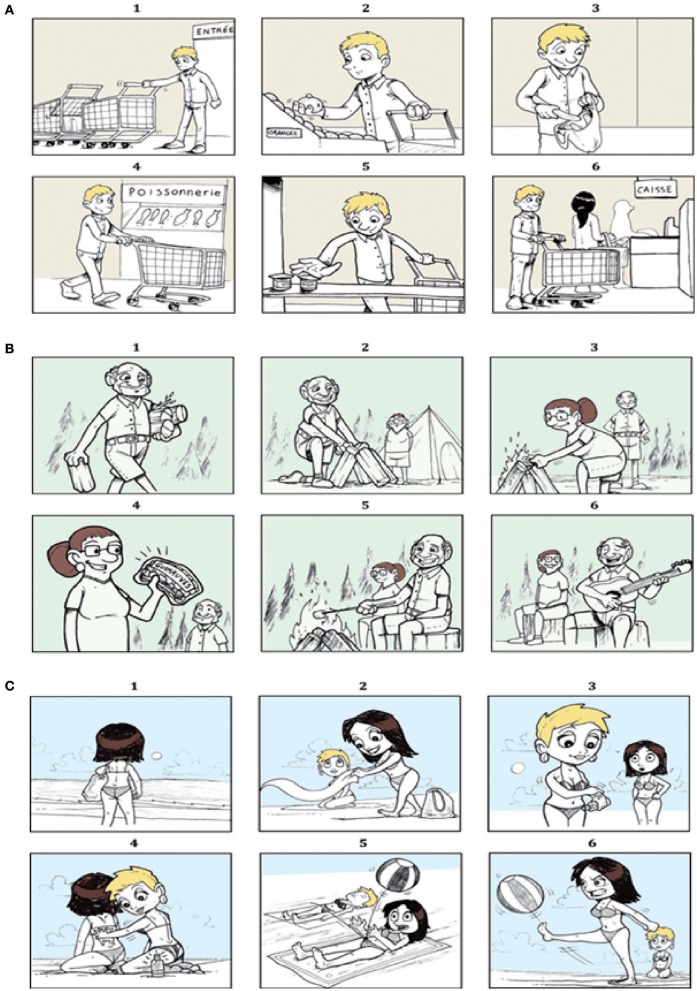
Examples of story sequences used in the storytelling in sequence task. **(A)** Complexity level-1 sequence (low referential complexity); **(B)** Complexity level-2 sequence (intermediate referential complexity); **(C)** Complexity level-3 sequence (high referential complexity).

The three levels of referential complexity are defined by the number of characters presented in the sequences (1 or 2 characters) and their gender (different or same gender; see Figure 1). Therefore, the three level-1 sequences, the referentially simplest, display only one character performing successive actions (Figure 1A); the three level-2 sequences are intermediate and display two characters of opposite sex (Figure 1B), which are not referentially ambiguous in gender; the three level-3 sequences, the most referentially complex, display 2 characters of the same gender (Figure 1C), therefore potentially ambiguous for reference.

##### Stages of discourse

The three stages of discourse were defined on the basis of the six images composing each sequence. The first image card always corresponded to the introduction of the first character, whereas for the subsequent image cards the discourse stages were prompted by manipulating the saliency of the characters displayed in the images according to the following contrast: The character is visually salient, in focus, if in the foreground and active in the image, whereas the character is visually non-salient, not in focus, if in the background and passive (Landragin, 2011). This manipulation allowed us to distinguish for each image, given its rank in the sequence, whether it corresponded to the stage of introduction (character 1), maintaining a character already in focus (character 1 or 2), or shift of the character who is in focus (character 1 or 2).

For the complexity level-1 sequences, only the introduction stage (Image 1), which introduces character 1 as a new referent, and the maintaining stage (from image 2–6), which maintains the focus on this character, are present in the sequence (see Figure 1A). The shift stage does not apply at this level as there is only one character in the sequence.

For the complexity level-2 and level-3 sequences, images 1 and 2 focus on character 1 (the visually salient, agent character), images 3 and 4 focus on character 2 (who becomes the visually salient, agent character), and images 5 and 6 focus again on character 1 (see Figures 1B,C). This manipulation of the relative salience of the two characters in the different images hence leads for each image, given its rank in the sequence, to the following stages of discourse. The introduction stage is also associated to Image 1, which corresponds to the introduction of character 1 (always displayed alone) as a new referent. The maintaining stage is associated to three images: Image 2 (maintaining “in focus” of character 1, previously in focus), Image 4 (maintaining “in focus” of character 2, previously in focus in Image 3), and Image 6 (maintaining “in focus” of character 1, previously in focus in Image 5). The shift stage is associated to the two remaining images: Image 3 (shift toward character 2, now in focus in this image but in the background in the previous one), and Image 5 (shift toward character 1, again “in focus” in this image but in the background in the previous one).

In summary, for the level-1 sequences, only the introduction (image 1) and maintaining (images 2–6) stages are involved and allow the analysis of reference markers/expressions. For the level-2 and level-3 sequences, on the other hand, the reference markers/expressions used to refer to the character in focus (the visually salient character) can be extracted and analyzed as introduction markers/expressions (image 1), maintaining markers/expressions (images 2, 4, 6) and shift markers/expressions (images 3 and 5).

#### Procedure

In order to make the storytelling in sequence task interactive, we used the referential communication paradigm, which reproduces a collaborative communication situation between two partners (Clark and Wilkes-Gibbs, 1986). Following the same procedure as that used in Achim et al. (2017) and Champagne-Lavau et al.'s study (2009) an opaque screen is placed between the two partners to prevent any non-verbal communication. In the current task, the participant plays the role of the speaker-narrator and thus receives the images of each sequence in the predetermined order. He/she is then invited to tell the story depicted in the sequence so that the addressee, who holds the same set of images but in a random order, can follow the story and place the images in the same predetermined order. The instructions given to the participants stressed that they had to tell a story, and not only describe the images one by one. Indeed, the major aim of this study being to analyze whether an adjustment of referential expressions takes place according to the discourse stages, it was important that the speakers-narrators produce a narrative, to obtain a potentially diversified range of reference markers.

During the task, the addressee could give some feedback to signal understanding (e.g., “*umh umh*,” “*ok*”) or to ask for clarifications in case of ambiguity (e.g., “*can you give me more details?*”). The procedure was repeated with the nine different sequences with the order randomized between participants.

Following the same procedure as that of Achim et al. (2017), the role of the addressee was held by a trained research assistant to standardize the feedback given for the different sequences. In particular, a strategy of “concealment” was developed to avoid that the participant, acting as a speaker-narrator, assume all sequences to be known from the addressee. The aim of this strategy was to make the participants believe that the addressee was also discovering the sequence of images for the first time. More specifically, the images were sealed in an envelope and the addressee explained that it was prepared by another person of the research team to prevent him from knowing the sequences. A pre-test demonstrated the success of this strategy with none of the 10 pilot subjects reporting suspecting that the addressee was familiar with the material used for the task. The pre-test also allowed us to adjust the instructions (forbid naming the characters or making them have a dialogue).

##### Predictability of the sequences

Even if all nine sequences depict short stories in which logical sequences of actions are presented (cf. Figure 1), a particular attention was given in sequence construction so that each sequence allows different plausible orderings. Our aim was to avoid the predictability of the sequence, which would have lessened the relevance from the interactive storytelling procedure. To ensure that our sequences allowed different plausible orderings, we conducted a pre-test with 15 subjects, simply asking them to order the six images of each of the nine sequences in order to make a story. The results confirmed that several orderings were possible, since three to four different orders were produced for each of the nine sequences.

#### Data processing

The interactions were tape-recorded and then transcribed verbatim. All stories were divided into fragments corresponding to the presentation of each of the six images composing each sequence. Within each fragment, we focused on the critical clause, the one referring to the character in focus (the character in the foreground and active in the image).

##### Extraction of referential expressions and coding of reference markers

Within each fragment the first referential expression produced to refer to the character in focus was extracted and, following Achim et al. (2017), we coded whether the referential expression began with: (a) an indefinite marker, (b) a definite marker, or (c) an unaccented pronoun (clitic pronouns and zero pronouns), as shown in Table 1. While indefinite markers and unaccented pronouns represent the two polar types of reference markers under marking the level of accessibility of the referents (i.e., low accessibility marking for indefinite markers and high accessibility marking for pronouns), the definite markers category was widely defined to include definite and possessive expressions as well as a few demonstratives and accented pronouns, that are expected to signal an intermediate level of accessibility (Ariel, 1990; Gundel et al., 1993; Cornish, 1999; Fossard et al., 2012). The inclusion of accented pronouns in the category of “medium accessibility” markers is due to the fact that these pronouns have indexical properties that are different from their unaccented counterparts. In French, in particular, they take a “strong” form (also called disjunctive) and, occupying a detached, separate position from the verb (i.e., dislocation), they are “capable of referring to entities which, though assumed to be recoverable by the addressee, are not the ones enjoying the highest degree of focus at the point of use” (Cornish, 1999, p. 63). This property distinguishes them from the unaccented pronouns that cannot assume this discourse function.

**Table 1:** Categories of reference markers.

**Category**	**Included in that category**	**Example**
Indefinite markers (IN)	Indefinite	“un garçon” (a boy)
Definite markers (D^+^)	Definite Possessive Demonstrative Accented pronouns (also called “disjunctive”)	“le garçon” (the boy) “son ami” (his friend) “ce garçon” (this/that boy) “… et donc lui, (il)…” (…and thus, HE…)
Unaccented pronouns (PR)	Clitic pronouns Zero pronouns	“il marche” (he walks) “…, marche… (…, walks…)

When several referential expressions were produced to refer to the character in focus in the same fragment, only the expression linking this character to the depicted action (i.e., the critical clause) was extracted. Most of the time, this expression corresponded to the first mention of the character in the fragment. For a few cases, however, it could correspond to the second mention, either because the first mention of the character in focus in the fragment was rather linked to a comment on the character's mood or personality, or because it did not individualize the character in focus in connection with the action depicted (i.e., plural references). For these cases, the reference linking the character to the depicted action occurred in a second mention, which was targeted for coding.

The cases for which the character in focus was only mentioned as part of a plural and not thereafter individualized were excluded from the analyses (31 occurrences across participants). We also excluded the cases where, in a given fragment, the character in focus was not mentioned (21 occurrences) as well as three other cases—two nouns without determiner and one generic pronoun “on” *(one)*—that did not fit within the categories of analyzed markers (see Table 1). In total, 3.4% of the whole data (55/1,620 references) was excluded.

Example 1 below presents a verbatim transcription^3^ showing which referential expressions were extracted and coded (underlined expressions) for each fragment (see the corresponding images in Figure 1C). The double slashes (//) indicate the divisions into fragments corresponding to the six images. The English translation of this verbatim is presented in brackets in italic font. Example 1:

“une jeune demoiselle au bord de la plage regarde la mer, ça lui plait, elle est en bikini (*a young lady* *by the beach looks out at the sea, she likes it, she is wearing a bikini*) // et *elle* étale sa serviette à côté de sa copine blonde *(and* *she* *spreads her towel out next to her blond friend)* // sa copine blonde décide de se passer de la crème solaire afin de ne pas brûler au soleil *(**her blond friend* *decides to put some sunscreen on herself so as not to burn under the sun)* // gentille comme elle est, elle étale de la crème sur le dos de sa copine également *(nice as she is*, *she* *also rubs some cream on her friend's back)* // elles sont toutes les deux tranquilles quand tout à coup la brune reçoit un ballon sur le ventre *(they are both quiet when suddenly* *the brunette* *receives a ball on the stomach)*// et énervée elle shoote dans ce ballon pour le renvoyer à l'expéditeur *(and upset* *she* *kicks this ball to send it back to the sender).”*

In this example, the first referential expression “une jeune demoiselle” *(a young lady)* used to refer to the character in focus in the first image was extracted and the reference marker used coded as an indefinite. At image 2, the expression “elle” *(she)*, coded as a pronoun, maintains the reference to this character whereas at image 3, the shift toward character 2 is indicated by the use of the expression “sa copine blonde” *(her blond friend)* coded as a definite. At image 4, the first expression used, the pronoun “elle” *(she)* in “gentille comme *elle* est” *(nice as she is)* is a comment about the character, not linked to the activity performed by the character; therefore, it is the second expression, the second pronoun “elle” *(she)* that links the character to the depicted action (critical clause), which was extracted. In a similar way, the first expression used at image 5 [the plural pronoun “elles” *(they)*] does not individualize the character in focus in connection with the action, unlike the second expression, “la brune” *(the brunette)*, which was thus extracted and coded as a definite. At image 6, the character in focus is maintained through the use of the expression “elle” *(she)*, extracted and coded as a pronoun.

As recently documented by Vogels et al. (2013), visual salience of a character in a picture—as measured by its position in the foreground or in the background of the scene—affects which character speakers are more likely to mention first, as the subject of their utterance. We assumed that it would also be the case for our data, but we verified this assumption by coding the extracted reference within the critical clause as subject or non-subject (i.e., object or other). This revealed that 29/1,565 (1.85%) of the extracted references, produced to refer to the character in focus, were made using a non-subject reference. In the vast majority of cases (98.15%), speakers referred to the character in focus as the subject of the critical clause.

Reliability of the coding was determined by having a research assistant code 10% of the transcribed verbatim (i.e., corresponding respectively to 9 stories of level-1 complexity, 10 stories of level-2, and 10 stories of level-3). This assistant was asked to identify the critical clauses and code the referential expressions both in terms of markers' categories (indefinite, definite markers and unaccented pronouns) and grammatical function (subject or non-subject). Cohen's Kappa statistic was k = 0.76, *p* < 0.001, showing a strong agreement between the two codings.

##### Coding of reference content

In line with our second objective, the information content provided in the NP was then coded. This further coding constitutes a distinct aspect of referential choices that is independent from the choice of reference markers, as it exclusively focuses on the content of NPs. Only the expressions introduced with a definite marker (D^+^ marker, see Table 1) were taken into account and further coded according to whether they contained or not a modification of the head noun (a “descriptive content,” Beun and Cremers, 1998). Two categories of reference content were considered: (a) definite expressions whose head noun is modified by a pre- and/or a post-modifier (modified definite expressions: mD^+^); and (b) simple definite expressions, with a basic canonical structure—determiner + head noun—without modification (unmodified definite expressions: uD^+^; Biber et al., 1999). The category of modified definite expressions included different types of modifications. Typically, scalar or color adjectives were used as premodifiers [“le vieux monsieur” (*the old man*)] or postmodifiers [“la fille blonde” (*the blond girl*)], whereas prepositional phrases, restrictive relative clauses and noun phrases in apposition, were always used as postmodifiers (see Table 2). It was not so rare either to find noun phrase structures with multiple modifiers (ex: “la fille brune_Adj_ du début_PrepPhrase_” (*the* brunette_Adj_ from the *beginning*_PrepPhrase_). Finally, accented pronouns (labeled as “empty-D^+^” or eD^+^) were the object of a third category of referential expression content (see Table 2). Although seldom produced in the narrations, the behavior of these pronouns, as we will see in the results, reveals a very systematic pattern of use.

**Table 2:** Categories of referential expression content.

**Category**	**Included in that category**	**Example**
Modified definite expressions (mD^+^)	Adjectives as pre-or postmodifier Prepositional phrase as postmodifier Restrictive relative clause as postmodifier Appositive NP as postmodifier Multiple modifiers (pre or post)	‘'Le vieux (monsieur) ‘' (the old man) ‘'La (fille) blonde” (the blond girl) ‘'sa copine avec le bikini rayé” (her friend with a striped bikini) ’'La fille qui a les cheveux blonds” (the girl who has blond hair) ‘'Son amie, la blonde,” La fille brune du début (the brunette from the beginning)
Unmodified definite expressions (uD^+^)	Simple NP without modifier	“la /cette fille” (the/ that or this girl) “son amie” (her friend)
Accented pronouns (eD^+^- empty-D^+^) (also called “disjunctive”)		“… et donc lui, (il)…” (…and thus, HE…)

#### Statistical analyses

Our analyses targeted specifically the references in subject position (i.e., excluding 1.85% of non-subject cases). The data was analyzed with logistic mixed models using the lmer function from the lme4 package (Bates et al., 2015b) implemented in R (R Core Team, 2015). For each category of reference marker presented in Table 1 (indefinite markers, definite markers or unaccented pronouns), we first computed the main model, which included the fixed effects of discourse stages (introduction, maintaining, and shift) and of complexity levels (low, intermediate, and high referential complexity), as well as the interaction between these two factors. The intercept for subjects was included as a random effect. For each category of reference marker, the central analyses were a series of likelihood ratio tests used to compare the main model with three simplified models that respectively excluded: (1) the interaction term; (2) the effect of discourse stages; (3) the effect of the complexity levels. These likelihood ratio tests allowed us to determine whether the interaction term, the discourse stages or the complexity levels significantly explained the responses.

Given the low number of items (i.e., three narrative sequences per complexity level), the intercept for items was not initially included as a random effect in the models. However, all analyses were repeated with this additional random effect and any difference in the observed pattern of results is presented in the results section (only applicable for the indefinite markers). The random slopes were not included in the models for two reasons: (1) to ensure that the fixed effects of interest were the only parameters that varied between the models to be compared with the log likelihood ratio tests; (2) to avoid overparametrized models that failed to properly converge (see Bates et al., 2015a).

To decompose the observed effects, the series of log likelihood ratio tests were followed by additional analyses contrasting the parameters from the global model for each pair of discourse stages, which was done separately for each complexity level (e.g., comparing introduction vs. maintaining at level 1, then level 2, etc.). *P*-values were adjusted for multiple testing based on the multivariate normal distribution of the test statistics (Hothorn et al., 2008).

For the indefinite markers, the logistic model estimation did not converge when including the maintaining stage, given that there were no instances of indefinite markers for this stage. The logistic mixed model analyses were thus repeated without the maintaining stage, and any effect of discourse stages for indefinite references would hence reflect a distinction between the introduction and the shift stage.

So as to be thorough, for the indefinite markers we then performed additional analyses to examine whether the maintaining stage significantly differed from the introduction stage or from the shift stage, separately for the different levels of referential complexity. We calculated paired-sample *t*-tests on the proportion of indefinite makers used for the maintaining stage vs. the introduction stage, as well as for the maintaining stage vs. the shift stage, but given the types of distributions of the data we did not rely on the t distribution to evaluate the *p*-values. Instead, we used sign permutations to estimate the empirical probability associated with the observed *t*-values (i.e., how often a *t*-value of this magnitude is observed by chance). More specifically, this was achieved by performing 1,000 random permutations in which a random sign, positive or negative, was given to the difference in probability between the two targeted discourse stages observed for each participant.

For our second objective, to investigate the effects of increasing the referential complexity, additional contrasts were performed on the estimates from the logistic mixed models to assess the effect of the number of characters (1 or 2 characters in the story i.e., level 1 vs. levels 2 and 3) and the effect of referential ambiguity between these characters (two characters of different, vs. same gender i.e., level 2 vs. 3). These analyses were done for the three types of reference markers (see Table 1), and additional regression models were computed to allow the same contrasts for each category of reference content presented in Table 2 (modified definite expressions, unmodified definite expressions, or accented pronouns). The additional models however did not converge for the unmodified definite expressions and the accented pronouns, and for these referential expressions we calculated paired-sampled *t*-tests to compare the proportions with which they were used for sequences with one vs. two characters (i.e., level 1 vs. levels 2 and 3) or with different or same gender (i.e., level 2 vs. 3). These comparisons were done separately at the different discourse stages, and the *p*-value of the obtained *t*-value was again empirically assessed with the permutation procedure described above.

## Results

**Figure 2:**
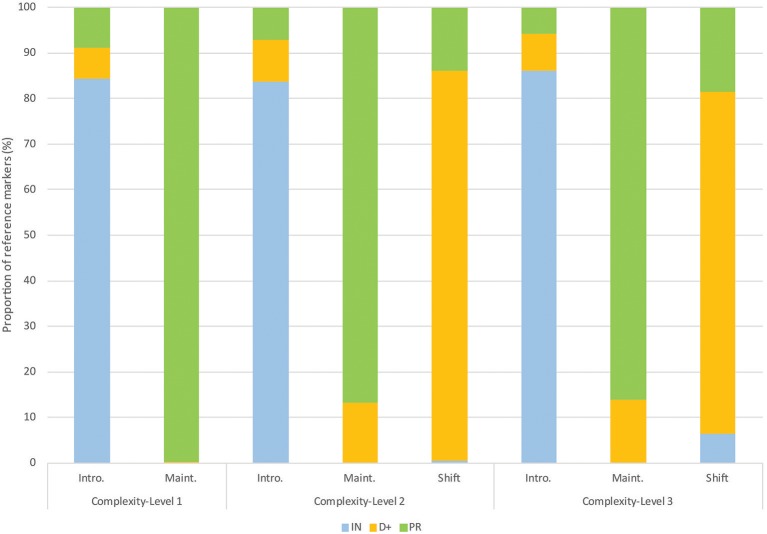
Proportion of each category of reference markers used for each discourse stage and each level of referential complexity-level of the stories. Each type of reference marker is presented with a distinct color.

The following sections present the results for each category of reference markers (indefinite markers, unaccented pronouns, and definite markers—see Tables 3–**5** and Figure 2) and for each category of referential expression content (modified definite expressions, unmodified definite expressions, and accented pronouns—see **Table 6**). While the analyses were performed for the three discourse stages, we also report the proportions at which the different reference markers were used for each individual image as Supplementary Material.

**Table 3:** Results for the adjustments in the use of indefinite markers (IN) at the different discourse stages for each of the three complexity levels of the stories.

	Introduction (Intro)^*^			**Maintaining (Maint)**			**Shift**			**Effects of discourse stages: Objective 1**		
	**nb. IN**	**nb. Other**	**% IN**	**nb. IN**	**nb. Other**	**% IN**	**nb. IN**	**nb. Other**	**% IN**	Intro^*^. vs. Maint.	Intro^*^. vs. Shift	**Shift vs. Maint**.
Complexity-Level 1	76	14	84.4	0	438	0	–	–	–	*t*_(29)_ = 14.3, *p* < 0.001	–	–
Complexity-Level 2	72	14	83.7	0	258	0	1	158	0.6	*t*_(29)_ = 14.3, *p* < 0.001	β = −9.4, *Z* = −7.5, *p* < 0.001	*t*_(29)_ = 1, N.S.
Complexity-Level 3	75	12	86.2	0	250	0	11	157	6.5	*t_(_*_29)_ = 15.3, *p* < 0.001	β = −7.2, *Z* = −8.7, *p* < 0.001	*t*_(29)_ = 3.6, *p* < 0.001
**Effects of complexity-Level: Objective 2**												
Complexity-Level 1 vs. 2 and 3	β = −0.16, *Z* = −0.31, N.S			–			–					
Complexity-Level 2 vs. 3	β = −0.36, *Z* = −0.61, N.S.			–			β = −2.5, *Z* = −2.36, N.S.					

*nb., number; IN, Indefinite markers; Other, all other types of markers*;

* *Indefinite markers are particularly expected at the introduction stage*.

### Indefinite references

The counts and proportions for the indefinite markers are presented in Table 3 for each discourse stage and each level of referential complexity (top-left). The comparisons of logistic mixed models (which here excluded the maintaining stage where there was no cases) revealed a significant effect of discourse stages [$X_{\left(2\right)}^{2}$ = 455.9, *p* < 0.001], a significant effect of referential complexity [$X_{\left(3\right)}^{2}$ = 10.4, *p* = 0.015] and a significant interaction between these two variables [$X_{\left(1\right)}^{2}$ = 3.99, *p* = 0.046].

Indefinite markers are mainly expected for the introduction stage, and as can be seen in Table 3 (right side of the table), participants used indefinite references significantly more often for the introduction stage than for the shift stage both for complexity level 2 and complexity level 3 sequences. Furthermore, our additional analyses (*t*-tests) allowed us to examine the comparisons with the maintaining stage, which revealed that participants used indefinite markers more often for the introduction stage than for the maintaining stage at all the levels of complexity. Participants also used indefinite markers more often for the shift stage than for the maintaining stage for sequences of complexity level 3 but not for complexity level 2.

When assessing the effect of referential complexity at the different discourse stages (bottom of Table 3), no significant effect emerged for either the introduction or the shift stage. No comparisons were made for the maintaining stage where there were no cases.

When the analyses were repeated including the random effect of items, the main effect of referential complexity and the interaction with the discourse stages no longer reached significance [respectively $X_{\left(3\right)}^{2}$ = 4.82, *p* = 0.185 and $X_{\left(1\right)}^{2}$ = 2.85, *p* = 0.092], but the main effect of discourse stages remained significant [$X_{\left(2\right)}^{2}$ = 461.6, *p* < 0.001] and the contrasts led to the same pattern of results as those presented in Table 3. A further examination of the data revealed that one of the level 3 sequences led to 10 of the 11 instance of IN observed for the shift stage.

### Unaccented pronouns

The counts and proportions for the unaccented pronouns are presented in Table 4 (top-left) for each discourse stage and each level of referential complexity. The comparisons of logistic mixed models revealed a significant effect of discourse stages [$X_{\left(5\right)}^{2}$ = 1028.5, *p* < 0.001], a significant effect of referential complexity [$X_{\left(5\right)}^{2}$ = 84.5, *p* < 0.001] and a significant interaction between these two variables [$X_{\left(3\right)}^{2}$ = 23.4, *p* < 0.001].

**Table 4:** Results for the adjustments in the use of pronouns (PR) at the different discourse stages for each of the three complexity levels of the stories.

	**Introduction (Intro)**			Maintaining (Maint)^*^			**Shift**			**Effects of discourse stages: Objective 1**		
	**nb. PR**	**nb. Other**	**% PR**	**nb. PR**	**nb. Other**	**% PR**	**nb. PR**	**nb. Other**	**% PR**	Intro. vs. Maint.^*^	**Intro. vs. Shift**	Shift vs. Maint.^*^
Complexity-Level 1	8	82	8.9	437	1	99.8	–	–	–	β = −8.7, *Z* = −8.1, *p* < 0.001	–	–
Complexity-Level 2	6	80	6.9	224	34	86.8	22	137	13.8	β = −4.7, *Z* = −9.9, *p* < 0.001	β = −0.8, *Z* = −1.6, N.S.	β = −3.9, *Z* = −12.6, *p* < 0.001
Complexity-Level 3	5	82	5.7	215	35	86	31	137	18.4	β = −4.9, *Z* = −9.5, *p* < 0.001	β = −1.4, *Z* = −2.69, *p* = 0.067	β = −3.5, *Z* = −12.2, *p* < 0.001
**Effects of complexity-Level: Objective2**												
Complexity-Level 1 vs. 2 and 3	β = 0.38, *Z = 0.78*, N.S			β = 4.2*, Z* = 4.23, *p* < 0.001			–					
Complexity-Level 2 vs. 3	β = 0.21*, Z* = 0.34, N.S.			β = 0.07, *Z = 0.25*, N.S.			β = −0.36, *Z* = −1.14, N.S.					

*nb., number; PR, (unaccented) pronouns; Other, all other types of markers*;

* *pronouns are particularly expected at the maintaining stage*.

Pronouns are mainly expected for the maintaining stage, and as can be seen in Table 4 (right side of the table), participants indeed used unaccented pronouns more often for the maintaining stage than for both the introduction stage and the shift stage at all the complexity levels where it applies (i.e., there is no shift stage for complexity level 1). In contrast, no significant difference emerged between the shift stage and the introduction stage regardless of the discourse stages.

When assessing the effect of referential complexity at the different discourse stages (bottom of Table 4), the only effect that emerged was for the maintaining stage, with more pronouns used for sequences with one character than for sequences with two characters (level 1 > levels 2 and 3). In contrast, there was no significant effect linked to the referential ambiguity between the two characters (i.e., different vs. same gender for levels 2 or 3, respectively).

### Definite references: reference markers

The counts and proportions for the definite markers are presented in Table 5 for each discourse stage and each level of referential complexity. The comparisons of logistic mixed models revealed a significant effect of discourse stages [$X_{\left(5\right)}^{2}$ = 493.1, *p* < 0.001], a significant effect of referential complexity [$X_{\left(5\right)}^{2}$ = 89.4, *p* < 0.001] and a significant interaction between these two variables [$X_{\left(3\right)}^{2}$ = 23.5, *p* < 0.001].

**Table 5:** Results for the adjustments in the use of definite markers (D^+^) at the different discourse stages for each of the three complexity levels of the stories.

	**Introduction (Intro)**			**Maintaining (Maint)**			Shift^*^			**Effects of discourse stages: Objective 1**		
	**nb.D^+^**	**nb.Other**	**% D^+^**	**nb.D^+^**	**nb.Other**	**% D^+^**	**Nb D^+^**	**nb.Other**	**% D^+^**	**Intro. vs. Maint**.	Intro. vs. Shift^*^	Shift^*^ vs. Maint.
Complexity-Level 1	8	84	6.7	1	437	0.2	–	–	–	β = 3.45, *Z* = 3.19, *p* = 0.015	–	–
Complexity-Level 2	8	78	9.3	34	224	13.2	136	23	85.5	β = −0.41, *Z* = −0.99, N.S.	β = −4.3, *Z* = −9.51, *p* < 0.001	β = 8, *Z* = 12.56, *p* < 0.001
Complexity-Level 3	7	80	8	35	215	14	126	42	75	β = −0.62, *Z* = −1.43, N.S.	β = −3.7, *Z* = −8.3, *p* < 0.001	β = 3.04, *Z* = 11.37, *p* < 0.001
**Effects of complexity-Level: Objective 2**												
Complexity-Level 1 vs. 2 and 3	β = −0.28*, Z* = −0.57, N.S.			β = −4.26, *Z* = −4.23, *p* < 0.001			–					
Complexity-Level 2 vs. 3	β = 0.16*, Z* = 0.29, N.S.			β = −0.06, *Z* = −0.22, N.S.			β = 0.74, *Z* = 2.54, N.S.					

*nb., number; D^+^, Definite markers; Other, all other types of markers*;

* *definite markers are particularly expected at the shift stage*.

Definite markers are mainly expected for the shift stage, and as can be seen in Table 5 (right side of the table), participants used definite markers more often for the shift stage than for the introduction stage, both for complexity level 2 and complexity level 3 sequences. Participants also used definite markers more often for the shift stage than for the maintaining stage, both for complexity level 2 and complexity level 3 sequences. For complexity level 1 a significant difference emerged between the introduction stage and the maintaining stage, with more definite markers for the introduction stage. The comparisons between the introduction stage and the maintaining stage were not significant for the complexity levels 2 or 3.

When assessing the effect of referential complexity at the different discourse stages (bottom of Table 5), the only significant effect that emerged was an effect of the number of characters observed only during the maintaining stage, with more definite markers for sequences with two characters compared to sequences with one character (levels 2 and 3 > level 1). No other effects of referential complexity reached statistical significance.

### Definite references: reference content

Table 6 presents the counts and the proportions for each category of referential expression content, namely modified definite expressions, unmodified definite expressions and accented pronouns. In addition, Table 6 also presents the effects of referential complexity manipulated in terms of the number of characters (level 1 vs. levels 2 and 3) and in terms of referential ambiguity (level 2 vs. 3) separately for each discourse stage.

**Table 6:** Results for the modulation in the use of reference content including: modified (mD^+^), unmodified (uD^+^), or “empty” (eD^+^) definite expressions (eD^+^ meaning “accented pronouns”) as a function of complexity levels of the stories for each of the three discourse stages.

**Modified (mD^+^)**	**Introduction**			**Maintaining**			**Shift**		
	**nb. mD^+^**	**nb. Other**	**%mD^+^**	**nb. mD^+^**	**nb. Other**	**%mD^+^**	**nb. mD^+^**	**nb. Other**	**%mD^+^**
Complexity-Level 1	3	87	3.3	1	437	0.2	–	–	–
Complexity-Level 2	5	81	5.8	4	254	1.5	22	137	13.8
Complexity-Level 3	4	83	4.6	15	235	6	82	86	48.8
**EFFECTS OF COMPLEXITY-LEVEL FOR mD**^+^									
Complexity-Level 1 vs. 2 and 3	β *= 0.46, Z* = 0.68, N.S.			β = −2.6, *Z* = −2.56, *p* = 0.096			–		
Complexity-Level 2 vs. 3	β = 0.24, *Z* = 0.35, N.S.			β = −1.4, *Z* = −2.48, N.S.			β = −1.9, *Z* = −6.63, *p* < 0.001		
**Unmodified (uD**^+^**)**	**Introduction**			**Maintaining**			**Shift**		
	**nb. uD**^+^	**nb. Other**	**%uD**^+^	**nb. uD**^+^	**nb.Other**	**%uD**^+^	**nb. uD**^+^	**nb. Other**	**%uD**^+^
Complexity-Level 1	3	87	3.3	0	438	0	–	–	–
Complexity-Level 2	3	83	3.5	29	229	11.2	101	58	63.5
Complexity-Level 3	3	84	3.4	20	230	8	44	124	26.2
**EFFECTS OF COMPLEXITY-LEVEL FOR uD**^+^									
Complexity-Level 1 vs. 2 and 3	*t*_(29)_ = 0, N.S.			*t*_(29)_ = 7.6, *p* < 0.001			–		
Complexity-Level 2 vs. 3	*t*_(29)_ = 0, N.S.			*t*_(29)_ = 1.2, N.S.			*t*_(29)_ = 6.5, *p* < 0.001		
**Empty(eD**^+^**)—accented pronouns**	**Introduction**			**Maintaining**			**Shift**		
	**nb. eD**^+^	**nb. Other**	**%eD**^+^	**nb. eD**^+^	**nb. Other**	**%eD**^+^	**nb. eD**^+^	**nb. Other**	**%eD**^+^
Complexity-Level 1	0	90	0	0	438	0	–	–	–
Complexity-Level 2	0	86	0	1	257	0.4	13	146	8.2
Complexity-Level 3	0	87	0	0	250	0	0	168	0
**EFFECTS OF COMPLEXITY-LEVEL FOR eD**^+^									
Complexity-Level 1 vs. 2 and 3	–			*t*_(29)_ = 1, N.S.			–		
Complexity-Level 2 vs. 3	–			*t*_(29)_ = 1, N.S.			*t*_(29)_ = 3.2, *p* < 0.003		

*nb., number; Other, all other types of markers*.

For the modified definite expressions (mD^+^), the only significant effect was observed for the shift stage, with greater use of the modified definite expressions when there was referential ambiguity between the two characters (level 3 > 2).

For the unmodified definite expressions (uD^+^), an effect of the number of characters emerged for the maintaining stage, with greater use of uD^+^ for stories with two characters (levels 2 and 3 > level 1). A significant effect of the referential ambiguity also emerged for the shift stage with greater use of uD^+^ when there was no referential ambiguity (level 2 > 3).

For the accented pronouns (eD^+^), the only significant effect was an effect of referential ambiguity again only observed for the shift stage, with more accented pronouns when there was no referential ambiguity (level 2 > 3).

## Discussion

This study used a new, collaborative storytelling in sequence task in order to examine how speakers adjust their referential choices depending on different narrative constraints implemented in the storytelling in sequence task. The narratives were produced based on sequences of six images from which we could identify specific discourse stages, corresponding to the introduction, maintaining or shift of the character in focus (i.e., the character that is visually salient and active in a given image). This manipulation was implemented within each sequence of images, with each image focusing more specifically on one character that was the most visually salient and active in the image. In addition, the task also included a manipulation of the referential complexity of the stories, which was implemented between the different sequences of images used to elicit the stories. This was achieved by manipulating both the number of characters (1 vs. 2) and, for the sequences in which there were two characters, their ambiguity in gender (different vs. same gender).

We hypothesized that our manipulations would influence the speakers' referential choices not only in terms of their choices of reference markers, which were expected to be strongly linked to the discourse stages, but also in terms of reference content. Reference content has been examined with tasks that involve presenting individual items, and assessing it in a narrative context represents an original contribution of the current study.

The results confirmed a strong effect of discourse stages on the choices of reference markers, such that indefinite markers were more often used to introduce the characters, unaccented pronouns were more often used to maintain the character in focus and definite markers were more often used to shift to a different character. As expected, this pattern was strong and observed for all levels of referential complexity.

The results also revealed some effects of the referential complexity of the stories on speakers' referential choices, both for the maintaining stage and the shift stage (but not for the introduction stage). For the maintaining stage, an effect of the number of characters was observed for the use of pronouns and of definite markers, such that pronouns were almost exclusively used to maintain the character in focus in stories with a single character), whereas definite references sometimes replaced the pronouns when there were two characters in the story (mainly unmodified definite expressions as revealed by the analyses targeting the reference content). For the shift stage, an effect of gender ambiguity was noted for all three categories of referential expression content, such that unmodified definite expressions and accented pronouns (“empty” D+) were more often used when the two characters were not ambiguous in gender whereas modified definite expressions were more often employed when there was referential ambiguity (characters of the same gender).

These results reveal that referential adjustments stand at various levels of granularity in discourse. Beyond the expected effects of discourse stages on choices of reference markers, the number of characters and their ambiguity in gender also modulated speaker's referential choices at specific discourse stages through adjustments of the reference content, likely to facilitate recognition of the intended referent by the addressee.

### Effects of discourse stages on adjustment of reference markers

The results of this study are in line with previous research on the use of referential expressions in narratives, which showed similar variations in the use of referential forms linked to different moments or discourse stages (e.g., Van der Lely, 1997; Colle et al., 2008; Hendriks et al., 2014; Contemori and Dussias, 2016). One interesting exception to this pattern has recently been reported by Hendriks et al. (2014), who observed a strong rate of definite references at a point in their stories at which they expected their second character to be maintained in focus (their “Maintain-2” condition; see also Contemori and Dussias, 2016: Experiment 2 for similar results with the same task and the same coding scheme). According to the authors, this unexpected pattern of results may be explained by the structure of the stories, in which the reference to the second character is maintained in a context of competition, due to the presence of the first (main) character in the discourse. For Hendriks et al. the presence of this other character could have led to the choice of a more explicit referential form (the NP) in order to avoid referential ambiguity.

Given that in our study the rate of pronouns was high across the different instances of the maintaining stage and predominant in all cases, including the maintaining of the second character in focus (Image 4; see the graphs in the Supplement for the “image by image” presentation of the results), it is interesting to further consider the distinctions between our task and the one developed by Hendriks et al. (2014). In our task, the discourse stages (introduction, maintaining and shift of the referent in focus) were defined on the basis of the relative visual salience and level of activity of the characters in each of the six images composing the sequences. As Vogels et al. (2013) previously showed, the visual salience of a character strongly influences the choice of the referent (especially if active in the foreground of the picture), guiding the speaker toward which character to refer to first, as the subject of the utterance. Through a manipulation of the visual salience and level of activity of the characters, our task allowed a clear identification of the maintaining stage, corresponding to the second consecutive time the character was in focus (i.e., maintained in focus), both in the images (character in the foreground and active in the picture) and in the discourse (since our analyses targeted specifically the references in subject position). In contrast, in the task proposed by Hendricks et al. the Maintain-2 position was defined as the second time the second referent was mentioned, without consideration of whether this referent was previously prominent (in subject position, for instance) or not when introduced in the discourse. There thus seems to be an important distinction between “*maintaining in the discourse*” vs. “*maintaining in the focus of attention*,” with the latter leading to use pronouns much more systematically than the former.

In the discussion of their paper, Hendriks et al. (2014) recognized that the speakers might not always have clearly established the second character as the new topic (i.e., as the referent in focus) before their Maintain-2 position. Hence, given the way it was defined, the Maintain-2 position proposed by Hendriks et al. (2014) could correspond more closely to what we defined as our first shift in focus toward the second character in our stories. This shift in focus occurred on our third image (in which the current focus is different from the prior focus) and we observed a strong use of definite references even if the second character had appeared in the background in the previous image (see the graphs in the Supplement for the “image by image” presentation of the results). This further highlights that it is not the mere prior presence of the character in the images or discourse, but the prior focus that influences the choice of specific reference markers. Essentially, in line with Vogels et al.'s study, our results show that the choice of the subject referent and the choice of the reference marker can be dissociated. While the visually foregrounded and active character is more likely to be chosen and referred to first, as the subject of the sentence, it is not more likely to be pronominalized: it depends on whether the referent was the subject of the previous sentence. For instance, when considering complexity levels 2 and 3, the character in focus at image 3 is different from that previously in focus at image 2, and the character in focus at image 5 is different of the character in focus at images 4. At images 3 and 5, the character in focus is very likely to be chosen as the subject of the sentence, but unlikely to be referred to with a pronoun, as it was not the subject of the previous sentence (i.e., not in focus in the previous picture) This defines a shift in focus, linked to the use of definite expressions.

Another important difference between the study by Hendriks et al. (2014) and the current study is that to elicit the narratives, they showed their images one at a time (i.e., successive presentation in a six-page storybook). With this presentation mode, speakers cannot globally apprehend the referential dynamics of the story (Trabasso and Nickels, 1992). Previous work by Canoz and Vion (1994) clearly demonstrated that the successive (picture by picture) or simultaneous (the entire sequence at once) presentation mode can influence reference choices. In their study, pronouns were used more often to maintain a referent in focus when the images on which the narrative is built were presented simultaneously than successively, with the inverse pattern observed for NPs. It is likely, thus, that the presentation mode of the pictures may have contributed to increase the rate of NPs in Hendriks et al.'s study (successive mode) and to reduce it in our study (simultaneous mode).

More subtle yet significant effects of discourse were also observed at specific discourse stages. Firstly, a greater use of indefinite references for the shift stage relative to the maintaining stage was observed specifically for the level-3 sequences (6.5% of the data, see Figure 2). As revealed following the analyses including the random effect of items, this pattern was largely driven by one of the narrative sequences of level 3. A closer look at these indefinite references revealed that the majority of them were produced at Image 3, corresponding to the first focus shift (see the graphs in the Supplement for the “image by image” presentation of the results). Some of these indefinite references were used to shift the focus of attention to a not previously mentioned referent (i.e., introducing character 2). For other cases, indefinite references were used to identify one of the characters as part of a unit, such as “un de ses camarades” (*one of his mates*). The structure of these expressions thus started with an indefinite pronoun “un-e” (*one*) followed by a prepositional phrase introduced by “de” (*of*). Interestingly, in this type of construction, the indefinite pronoun “un” (*one*) is anaphoric, as it refers to an entity that is part of an already known or accessible set of referents (Kleiber, 2001).

Secondly, a greater use of definite references for the introduction stage (6.7%) relative to the maintaining stage (0.2%) was observed specifically for level-1 sequences (stories with only one character). The almost exclusive use of pronouns at the maintaining stage for the level-1 sequences (see Figure 2) shows how strong this effect gets when the focus is not only already established but also entirely clear and predictable.

### Effects of referential complexity of the stories on referential choices based on discourse stages

Interestingly, the effects of the referential complexity of the stories on speaker's referential choices varied depending on the discourse stages.

For the maintaining stage, the only aspect of complexity that showed a significant effect was the number of characters (1 vs. 2), resulting in both a decrease in the use of pronouns (99.8 vs. 86.4%, respectively) and an increase in the use of definite markers (0.2 vs. 13.6%, respectively, mainly unmodified expressions, see the analyses targeting the reference content, Table 6). This result is in line with previous research, which showed that the presence of another character in the discourse and/or the visual context decreases the use of pronouns in favor of full NPs (e.g., Arnold and Griffin, 2007; Fukumura et al., 2010; Contemori and Dussias, 2016: Experiment 1). Since Arnold and Griffin's study (2007), these findings are interpreted as indicating a competition effect between two similar entities (two characters), resulting in a decrease of the level of activation of the most accessible referent, leading the speaker to opt for a more informative expression. However, it is remarkable that, in these studies, the decrease in pronoun rate is quite strong, dropping by 45–60% depending on the studies (Arnold and Griffin, 2007; Fukumura et al., 2010; Contemori and Dussias, 2016), between the single-character condition and the two-character condition. For instance, in Arnold and Griffin's study (2007), a little <20% of pronouns is used to maintain the referent in focus when a second character of different gender is present, a relatively low rate of pronouns. It also seems that, at least in some conditions, the rate of pronouns can drop even further (e.g., around 10% in Contemori and Dussias, 2016) when the second character has the same gender as the referent. At least two studies manipulated the referents in terms of gender ambiguity (cf. Fukumura et al., 2010; Contemori and Dussias, 2016), and the difference between maintaining the referent in the context of two different gender characters vs. two same gender characters was significant in these studies, indicating a decrease in the use of pronouns when the two characters are ambiguous in gender. In the current study, no gender ambiguity effect was found at the maintaining stage, either for pronouns or for definite markers.

Although the results of the current study also indicate a competition effect, with a decrease in the use of pronouns to maintain the referent in focus when a second character is present, the drop is modest, by 13.4%, and importantly, the rate of pronouns remains quite high (86.4% on average) compared to the rates of the studies presented above. Why, then, did participants in the current study continue to use pronouns massively in the presence of a second character whereas, in the other studies, this rate was significantly diminished, hardly exceeding 20%? We propose that this discrepancy might be due to the type of tasks used. Indeed, in all the aforementioned studies, a paradigm of story completion was used, in which participants were asked to repeat or read aloud a context sentence describing a first picture, and then to complete the story by generating the next sentence based on a second picture. In contrast, our storytelling in sequence task emphasized the need for a narrative, such that the participant was responsible for producing the full story, and not only the last sentence. This is an important point as pronouns have a function as continuity markers, serving to indicate to the addressee that the focus on a given referent is ongoing (Kleiber, 1994; Cornish, 1999). This major discursive function of the pronoun, which allows including the referent within a larger narrative context, and not only to signal a salient discourse status, was probably toned down in the story completion experiments. Therefore, participants would not project themselves into a narrative activity, but rather perform a picture description, which could in part explain the low production of pronouns in previous story completion studies. In contrast, other studies that used storytelling tasks (Colle et al., 2008; Achim et al., 2017) also reported high rate of pronouns for the maintaining stage, consistent with the suggestion that pronouns mark continuity within a wider discourse context.

Regarding the shift stage, an effect of gender ambiguity emerged for all three categories of referential expression content.

Firstly, speakers produced more informative expressions (i.e., modified definite expressions) more often when there was gender ambiguity between the two characters than when there was no ambiguity (48.8 vs. 13.8%, respectively). These observations are consistent with findings of previous studies that showed that speakers adjust the content of their referential expressions by extending the information provided in the NP, particularly when another referent of the same type is present (e.g., Brown-Schmidt and Tanenhaus, 2006; Engelhardt et al., 2006; Koolen et al., 2011). Whereas these studies used target identification tasks only, the current study shows that these effects also apply in a narrative context, and more specifically at the shift stage, indicating that speakers continue to adjust their referential choices as the discourse unfolds. These adjustments, which demonstrate a high sensitivity of the speakers to the referential ambiguity of level-3 sequences, also make the retrieval of the targeted referent easier for the partner. Hendricks et al. had suggested that “topic shift crucially requires speakers to take into account the listener's perspective” (p. 395, Hendriks et al., 2014), which would result in the use of definite NPs. Our results support this idea, and further suggest that, when the referential context is ambiguous (with two characters of same gender), speakers tend to further specify the content of their definite expressions by adding modifiers to the head noun of the definite NP (e.g., “the blond girl” vs. “the girl”).

Secondly, results of the current study also indicated that speakers more often produced less informative expressions (unmodified definite expressions) and accented pronouns when there was no gender ambiguity (63.5 and 8.2%, respectively) than when there was gender ambiguity (26.2 and 0%, respectively). These findings suggest that speakers are also well able to adjust their referential expressions by diminishing the informational content when the referential complexity of the stories decreases. The use of accented pronouns at the shift stage further reveals a very systematic pattern of use. Albeit infrequent, these pronouns (also called stressed pronouns) were exclusively produced during level-2 sequences, in which the two characters are not ambiguous in gender (e.g., “and *HE* puts marshmallows on the barbecue,” for image 5 in Figure 1B). A referentially unambiguous context hence seems needed to use this type of pronouns to bring a referent into focus (i.e., the shift stage). These results thus support the suggestion of Gundel that “stressed personal pronouns typically imply the referent is not in focus, i.e., they imply a focus shift” (p. 186, Gundel, 1998).

Overall, the observed effects of referential complexity in this study suggest quite flexible referential behavior by the speakers. Those adjustments arising during narrative could be different from those arising during target identification tasks. Several target identification studies emphasized that speakers often overspecify their references and provide more information than is strictly necessary for identification (Engelhardt et al., 2006; Arts et al., 2011; Koolen et al., 2011). Our observations show that, at least in a narrative context in which speakers have themselves generated the previous references, overspecification does not seem to be as frequent as in target identification tasks, probably as narrative requirements are wider than identifying a referent only. This point would definitely deserve further investigation.

## Conclusion

This study makes a significant contribution to the study of the factors that affect the use of referential expressions in discourse. It presents a new, collaborative storytelling in sequence task, which goes beyond previous tasks by introducing a standardized procedure to analyze referential choices of speakers when they refer to more or less accessible referents (based on three discourse stages) in increasingly complex referential contexts. In particular, the fact that in this task, the discourse stages—introduction, maintaining and shift of the referent in focus—were defined on the basis of the relative visual salience and level of activity of the characters in each of the six images composing the sequences, represents an important advance, greatly facilitating discourse analysis and the extraction of referential expressions. In addition, this task and its accompanying coding procedure allow the analysis of the referential choices not only in terms of reference makers (indefinite markers, definite markers, pronouns), but also in terms of reference content (i.e., modified vs. unmodified NPs), which constitutes a very original way to analyze referential adjustment processes as a function of the referential complexity of the stories.

## Author contributions

MF, AMA, and MC-L: Conceived and designed the study; SG and LR-V: Collected and transcribed the data and contributed to the coding; AMA and AB: Ran the analyses; MF and AMA: Interpreted the data and wrote the manuscript; All authors revised the work and approved the final version.

### Conflict of interest statement

The authors declare that the research was conducted in the absence of any commercial or financial relationships that could be construed as a potential conflict of interest.

## Footnotes

^1^Note that, as stories contain six pictures and only five discourse positions are analyzed, there is not a strict matching between pictures and discourse positions.

^2^In Hendriks et al. (2014), the maintaining of character 2, which corresponds to the fourth discourse position, appears to be matched with the presentation of the fourth or fifth pictures in the story, but the authors are silent on this.

^3^The feedback given by the addressee (umh umh, OK…) is not transcribed here.
