# Supplementary figures and images for: Referential Choices in a Collaborative Storytelling Task: Discourse Stages and Referential Complexity Matter
Source: Front Psychol. 2018 Feb 20;9:176. doi: 10.3389/fpsyg.2018.00176 (PMC5826302; doi:10.3389/fpsyg.2018.00176)

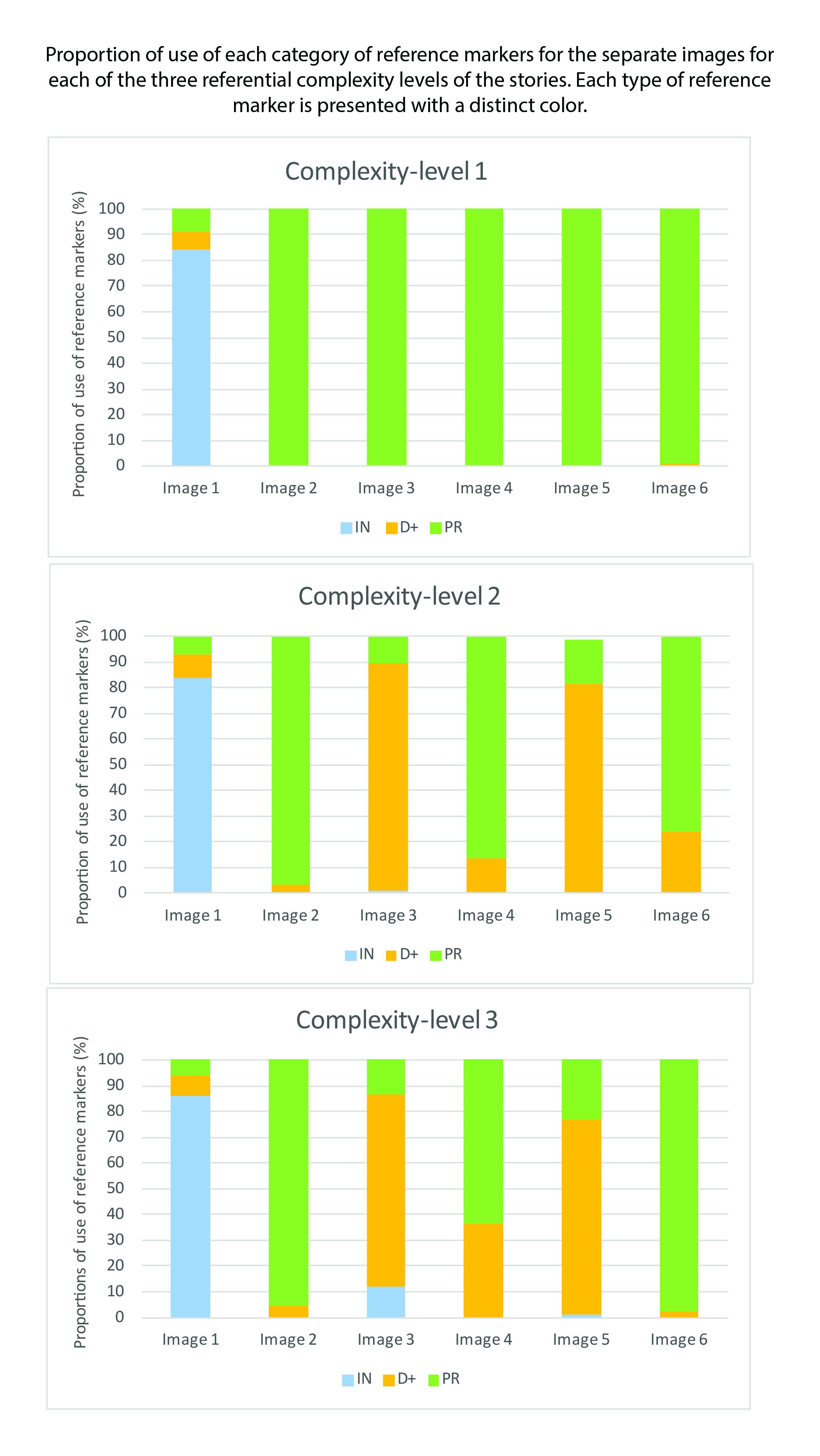

Supplement: Supplementary file 1 [file Image1.JPEG]
